# Anesthetic Management for the Retrieval of Retained Intrathoracic Foreign Bodies Adjacent to Critical Mediastinal Structures Using One-Lung Ventilation With a Bronchial Blocker: A Report of Two Cases

**DOI:** 10.7759/cureus.110538

**Published:** 2026-06-09

**Authors:** Sonali Gupta, Nistha Malik, Garima Choudhary, Abhishek Singh

**Affiliations:** 1 Anesthesiology, All India Institute of Medical Sciences, New Delhi, IND

**Keywords:** bronchial blocker, difficult airway management, fiberoptic bronchoscopy, metallic foreign body, one-lung ventilation

## Abstract

Retained intrathoracic metallic foreign bodies (FBs) following penetrating chest trauma represent a challenging surgical emergency, particularly when situated in proximity to critical mediastinal structures. Anesthetic management in such cases demands meticulous airway planning, expertise in lung isolation techniques, and vigilant anticipation of physiological changes during one-lung ventilation. We describe two adult men with penetrating chest trauma and retained metallic FBs adjacent to critical mediastinal structures, both managed under general anesthesia with left lung isolation using a bronchial blocker (BB). The first patient, a 40-year-old factory worker, sustained an injury from an iron nail ejected from an industrial sewing machine, resulting in a retained left paratracheal FB and ipsilateral hemopneumothorax. Despite preexisting chronic obstructive pulmonary disease, the nail was successfully retrieved via video-assisted thoracoscopic surgery. The second patient, a 34-year-old man, sustained a gunshot wound with a bullet lodged in the superior mediastinum, abutting the left brachiocephalic vein; the bullet was successfully retrieved via open thoracotomy. In both cases, the airway was secured via video laryngoscopy-guided tracheal intubation with a single-lumen tube, followed by fiberoptic-guided placement of the BB in the left main bronchus. Both FBs were retrieved without injury to adjacent structures. Both patients underwent planned postoperative mechanical ventilation and subsequently recovered uneventfully. These cases highlight the utility of a sequential airway strategy, in which the airway is secured first, and a bronchial blocker is subsequently deployed to facilitate the safe retrieval of deeply situated intrathoracic FBs across differing surgical approaches. They underscore the necessity of individualized anesthetic planning, tailored lung-isolation device selection, and vigilant intraoperative monitoring in complex thoracic trauma.

## Introduction

Chest trauma is the second leading cause of unintentional traumatic injury globally, with penetrating thoracic injuries (PTIs) carrying substantial morbidity and mortality [1,2]. Retained intrathoracic foreign bodies (FBs) following PTIs pose distinct risks beyond the acute phase, including empyema, hemothorax, tracheobronchial fistula, aortic erosion, and mediastinitis if not retrieved promptly [1,3]. The anatomical location of the FBs is the primary determinant of both the acuity of presentation and the complexity of surgical retrieval [4]. Deeply situated FBs, particularly those adjacent to the tracheobronchial tree, great vessels, or cardiac structures, require operative intervention under general anesthesia with reliable lung isolation [5]. While video-assisted thoracoscopic surgery (VATS) offers superior visualization and reduced postoperative morbidity, open thoracotomy is reserved for FBs requiring wide exposure or immediate vascular control [4,6].

One-lung ventilation (OLV) is the cornerstone of thoracic anesthesia. Bronchial blockers (BBs), such as the Arndt BB (Cook Medical, Bloomington, Indiana), are placed through a standard single-lumen endotracheal tube (ETT) under fiberoptic bronchoscopy (FOB) guidance and are particularly suited to trauma settings where airway security must be established as an initial priority before lung isolation is applied [7,8]. We report two cases of retained intrathoracic metallic FBs in proximity to critical mediastinal structures, highlighting the role of individualized anesthetic planning and lung isolation strategy in complex penetrating thoracic trauma.

## Case presentation

Case one

A 40-year-old male factory worker (BMI 24.8 kg/m²) presented following accidental PTI from a high-velocity iron nail ejected from an industrial sewing machine. He had initially been evaluated at a peripheral hospital, where supportive management was instituted prior to transfer. On arrival, he reported left-sided chest pain and dyspnea, with peripheral oxygen saturation of 96% on supplemental oxygen and reduced air entry over the left hemithorax. Chest radiography demonstrated left-sided hemopneumothorax with a retained radiopaque FB in the left paratracheal region and identified a malpositioned intercostal drain (ICD) inserted at the referring facility, with its tip directed toward the diaphragm rather than the pleural apex, resulting in inadequate drainage (Figure 1).

**Figure 1:**
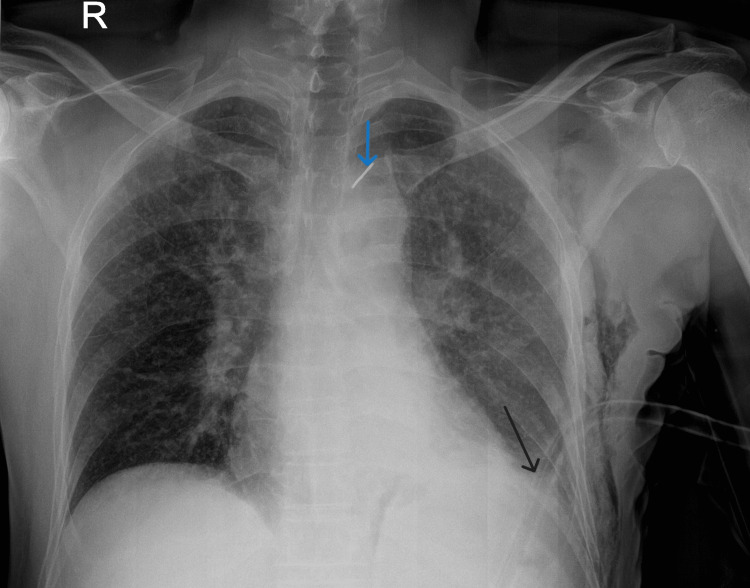
Anteroposterior chest radiograph demonstrating a retained radiopaque foreign body in the left paratracheal region (blue arrow) and a malpositioned intercostal drain with its tip directed toward the diaphragm rather than the pleural apex (black arrow).

High-resolution computed tomography (HRCT) of the chest corroborated the presence of a left paratracheal metallic FB consistent with an iron nail (Figure 2). 

**Figure 2:**
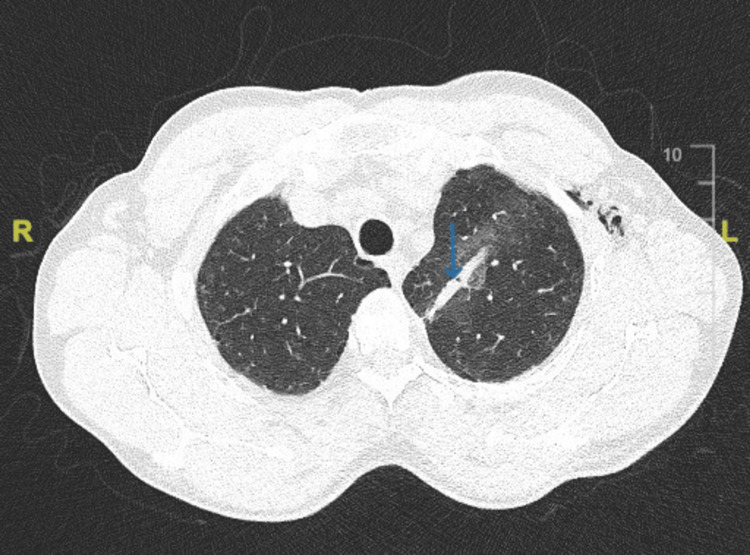
Axial section of high-resolution computed tomography of the chest showing a linear metallic foreign body (blue arrow) lodged in the left paratracheal region.

The malpositioned ICD was removed and replaced with a fresh ICD, which yielded 180 mL of altered blood as the initial output, and the drain position was confirmed on chest radiography. Preanesthetic evaluation revealed loose lower incisors and a history of chronic obstructive pulmonary disease (COPD). Airway assessment demonstrated a modified Mallampati (MMP) grade II airway, mouth opening of 4 cm, a thyromental distance of 6 cm, unrestricted neck movements, and no previous history of difficult intubation. Standard monitoring, right radial arterial cannulation for continuous blood pressure and arterial blood gas (ABG) monitoring, and right internal jugular vein (IJV) central venous catheterization were established. Following preoxygenation with 100% oxygen for three minutes, anesthesia was induced with intravenous fentanyl (2 µg/kg), propofol (2 mg/kg), and rocuronium (0.9 mg/kg). The trachea was intubated with an 8.5-mm cuffed ETT using a C-MAC® video laryngoscope (Karl Storz, Tuttlingen, Germany) on the first attempt, with care to avoid contact with the loose incisors. A 9-Fr Arndt BB was then advanced through the ETT under direct FOB guidance and positioned in the left main bronchus, with cuff occlusion confirmed endoscopically (Figure 3).

**Figure 3:**
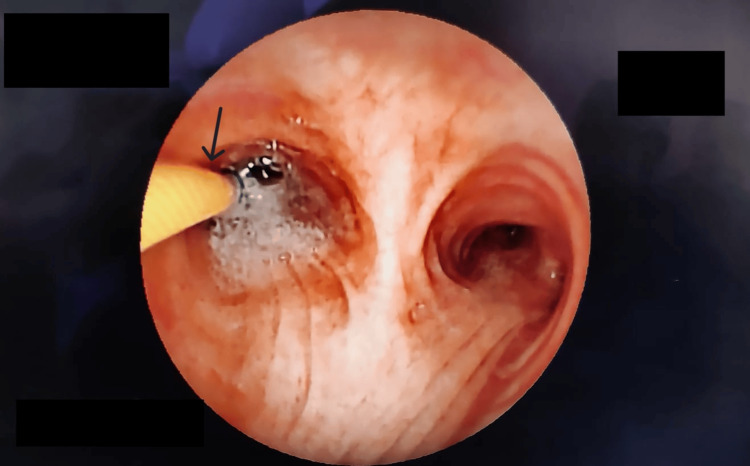
Fiberoptic bronchoscope image confirming correct placement of the 9-Fr Arndt bronchial blocker in the left main bronchus (black arrow).

OLV was conducted with a tidal volume of 7 mL/kg ideal body weight, positive end-expiratory pressure (PEEP) of 5 cmH₂O, and peak airway pressures of 22-25 cmH₂O. Serial intraoperative ABG analysis during OLV demonstrated satisfactory oxygenation and ventilation, with a PaO₂ of 136 mmHg and PaCO₂ of 42 mmHg on an FiO₂ of 0.5, confirming adequate gas exchange on the ventilated right lung despite preexisting COPD. The patient remained hemodynamically stable throughout the surgery. Under VATS, the iron nail (measuring approximately 1.5 cm in length) was retrieved without injury to adjacent vascular, esophageal, or airway structures (Figure 4).

**Figure 4:**
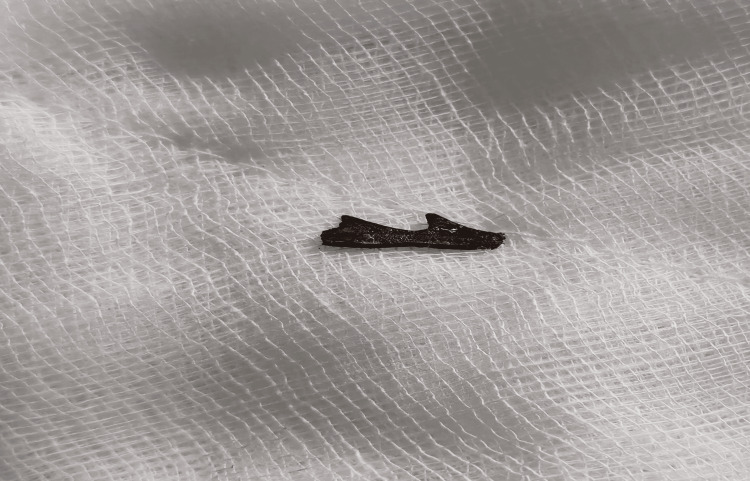
The retrieved iron nail following successful extraction from the left pleural cavity.

The patient was transferred to the intensive care unit (ICU) for planned postoperative mechanical ventilation and recovered uneventfully.

Case two

A 34-year-old man (BMI 30.8 kg/m²) was referred following a gunshot wound to the chest. He was hemodynamically stable with no neurological deficit. He complained of pain in the left side of the chest. Chest radiography demonstrated a bullet in the upper left hemithorax (Figure 5), and contrast-enhanced computed tomography (CECT) of the chest revealed pneumomediastinum with the FB (bullet) within the superior mediastinum abutting the left brachiocephalic vein (Figure 6).

**Figure 5:**
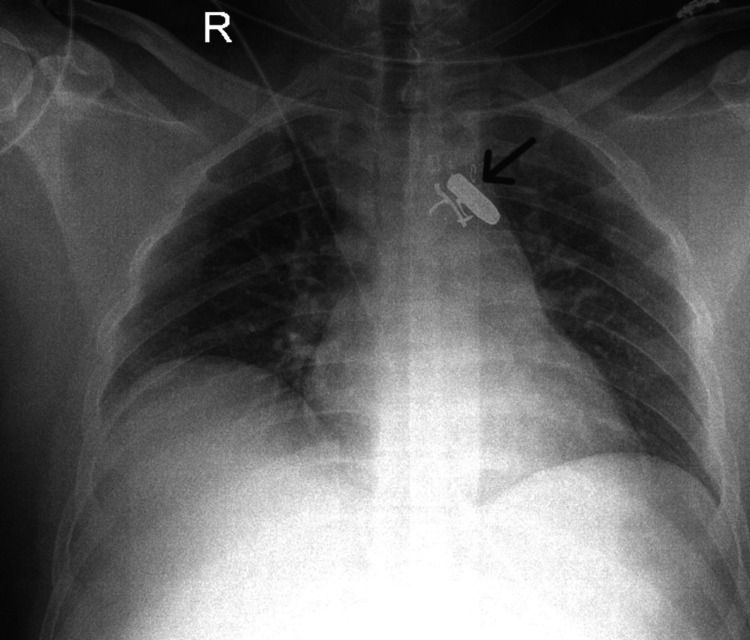
Anteroposterior chest radiograph demonstrating a radiopaque foreign body (black arrow) in the upper left hemithorax.

**Figure 6:**
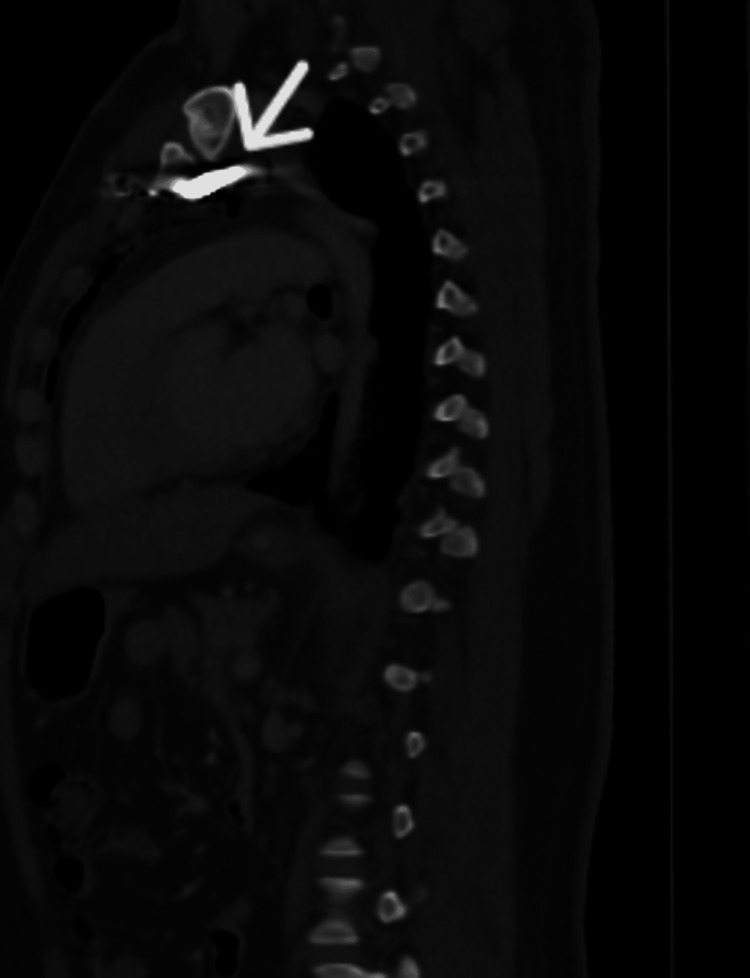
Contrast-enhanced computed tomography of the chest (sagittal section) demonstrating a metallic foreign body (bullet) in the superior mediastinum (white arrow), in close apposition to the left brachiocephalic vein.

Given the proximity to a major vascular structure, open thoracotomy was scheduled to ensure adequate exposure and readiness for vascular control. Preoperative airway examination revealed an MMP Grade IV airway, mouth opening of 3.5 cm, and a thyromental distance of 5 cm, with restricted neck flexion attributable to obesity.

Standard monitoring, right radial arterial cannulation, and right IJV access were established. Following three minutes of preoxygenation, anesthesia was induced with intravenous fentanyl (2 µg/kg lean body weight), propofol (2 mg/kg lean body weight), and rocuronium (0.6 mg/kg ideal body weight). Anticipating a difficult airway, the trachea was intubated with an 8.0-mm cuffed ETT using a C-MAC® video laryngoscope on the first attempt. A 9-Fr Arndt BB was placed under FOB guidance in the left main bronchus (Figure 7).

**Figure 7:**
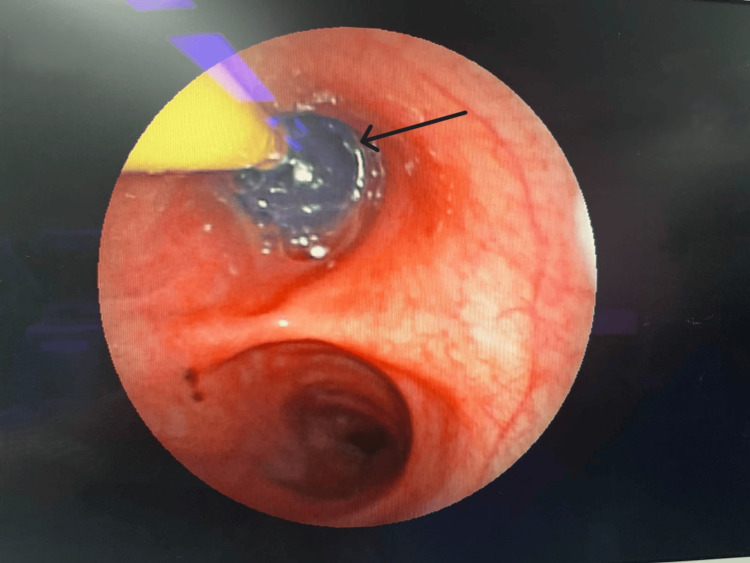
Fiberoptic bronchoscope image confirming placement of the 9-Fr Arndt bronchial blocker in the left main bronchus (black arrow).

OLV was conducted with a tidal volume of 7 mL/kg ideal body weight, PEEP of 5 cmH₂O, an initial FiO₂ of 0.5, and peak airway pressures of 27-29 cmH₂O. Oxygen saturation was maintained above 95% throughout without the need to increase FiO₂ or apply additional recruitment maneuvers. Serial intraoperative ABG analysis during OLV revealed a PaO₂ of 126 mmHg and PaCO₂ of 45 mmHg, reflecting adequate oxygenation and ventilation despite the increased atelectasis risk associated with obesity and reduced functional residual capacity. The patient remained hemodynamically stable throughout the surgery. The bullet was successfully retrieved from the superior mediastinum without major vascular breach (Figure 8); small fragments embedded within the sternum were left in situ, given the risk of additional vascular injury. The patient was transferred to the ICU for elective postoperative mechanical ventilation, extubated the following day, and discharged one week later without complications.

**Figure 8:**
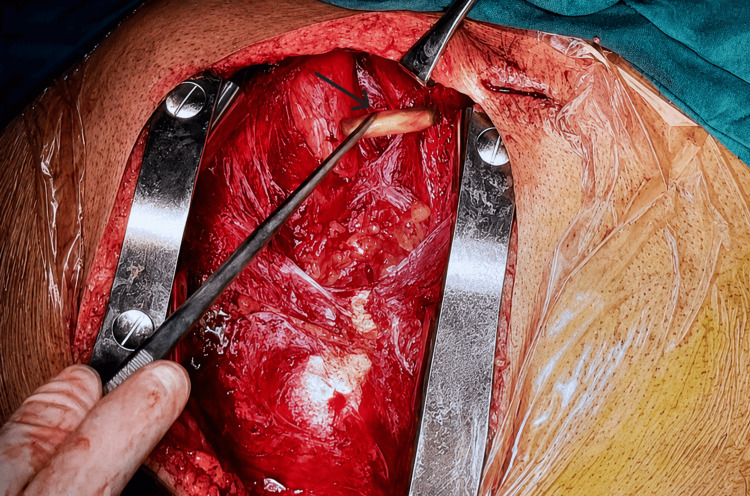
Intraoperative image from open thoracotomy showing the retrieved bullet (black arrow) following successful extraction from the superior mediastinum without major vascular breach.

## Discussion

These two cases illustrate the anesthetic management of retained intrathoracic metallic FBs in high-risk anatomical locations, emphasizing the role of OLV via bronchial blocker in trauma patients and the importance of individualized, stepwise surgical and anesthetic planning. Both patients were managed through a common airway strategy: C-MAC video laryngoscopy-guided tracheal intubation with a single-lumen ETT, followed by FOB-guided Arndt BB placement for reliable left lung isolation, despite differences in mechanism, FB location, comorbidity, and surgical approach.

Retained intrathoracic FBs following PTIs are predominantly encountered in adults, in contrast to foreign body aspiration, which is more prevalent in the pediatric population [3,5]. The clinical significance of a retained FB is primarily determined by its anatomical position: paratracheal and mediastinal objects may present acutely with hemothorax, pneumothorax, or pericardial tamponade, and carry a risk of delayed complications including empyema, tracheobronchial fistula, and mediastinitis if not retrieved promptly [1,3]. In the first case, the left paratracheal position of the iron nail in close proximity to the left main bronchus constituted a high-risk anatomical scenario demanding urgent operative retrieval. In the second, the bullet's relationship to the left brachiocephalic vein in the superior mediastinum carried a risk of major venous hemorrhage and was the principal determinant of both the urgency and operative approach.

The choice of operative approach is individualized to the FB's anatomical location and its relationship to vital structures. VATS has emerged as the preferred approach for many retained thoracic FBs, offering superior visualization, reduced postoperative pain, shorter hospital stay, and lower respiratory morbidity [4,6]. These advantages are particularly relevant in patients with preexisting COPD, in whom thoracotomy may precipitate prolonged ventilatory dependence; accordingly, the paratracheal iron nail was retrieved thoracoscopically without conversion. In the second case, the bullet's close relationship with the left brachiocephalic vein in the superior mediastinum favored open thoracotomy, which affords direct exposure and immediate readiness for proximal and distal vascular control should venous injury occur during retrieval [4].

Double-lumen tubes (DLTs) remain the gold standard for OLV in elective thoracic surgery owing to the ability to suction each lung independently and to switch rapidly between OLV and two-lung ventilation [7,8]. However, DLT placement in a trauma setting carries specific hazards: the bulkier design increases the risk of dental injury, tracheobronchial trauma, and malposition in a time-pressured environment, and in patients with anticipated or actual difficult airway, a DLT may compound the risk of failed intubation [7]. The Arndt BB, placed through an existing single-lumen ETT under FOB guidance, offered a clinically superior alternative in both patients [7,8]. This approach permitted the primary airway to be secured first with a conventional ETT under video laryngoscopy, with lung isolation then applied as a deliberate second step. The advantage was especially salient in the second case, in which an MMP Grade IV airway and obesity predicted difficult laryngoscopy; securing the trachea first with a single-lumen ETT under C-MAC video laryngoscopy, and only then introducing the BB, avoided the additional hazard of placing a bulky DLT in a difficult airway. FOB guidance was indispensable for accurate placement in both cases, providing real-time confirmation of cuff position within the left main bronchus [7].

Despite their advantages in trauma patients, BBs have several limitations. Compared with DLTs, BBs may result in slower lung deflation because of the smaller lumen of the blocker channel, which can delay surgical exposure in urgent situations. Suctioning and clearance of secretions from the isolated lung are also limited, making bronchoalveolar sampling or lung toilet more challenging. In addition, BBs are more susceptible to intraoperative displacement, particularly during patient repositioning or surgical manipulation, potentially leading to loss of lung isolation. Therefore, periodic confirmation of the BB position using FOB is recommended. In both cases reported here, these limitations were mitigated by meticulous FOB-guided placement, secure fixation of the BB at the connector, and continuous intraoperative monitoring; no displacement or loss of lung isolation was encountered in either patient.

The two cases represent distinct but overlapping risk profiles for OLV, involving COPD in the first patient and obesity in the second. COPD increases the risk of auto-PEEP, barotrauma, and hypoxemia during OLV due to airflow obstruction and reduced baseline gas exchange reserve, while obesity reduces functional residual capacity and promotes atelectasis, similarly predisposing to desaturation during OLV [8,9]. A lung-protective ventilation strategy was employed in both patients, with tidal volumes of 6-7 mL/kg ideal body weight, peak airway pressures below 30 cmH₂O, and PEEP applied to the ventilated lung to maintain alveolar recruitment [9]. Continuous ABG monitoring via the radial arterial cannula permitted real-time assessment and early intervention in both cases. Elective postoperative mechanical ventilation was planned in both patients in view of the combined burden of reduced respiratory reserve, the physiological stress of thoracic surgery, and the anticipated pleural and mediastinal inflammatory response. Each patient was electively ventilated in the ICU, recovered uneventfully, and was discharged without complications.

## Conclusions

We report the successful anesthetic management of two adults with retained intrathoracic metallic FBs adjacent to critical mediastinal structures. Both were managed through a common airway strategy: C-MAC video laryngoscopy for atraumatic tracheal intubation, followed by FOB-guided Arndt BB placement for reliable left lung isolation and lung-protective OLV tailored to each patient’s respiratory reserve. These cases reinforce that individualized anesthetic planning, with appropriate selection of the lung isolation device and operative approach, is the cornerstone of safe management in complex thoracic trauma.
